# Supplementary material for: Inferring bacterial transmission dynamics using deep sequencing genomic surveillance data
Source: Nat Commun. 2023 Oct 31;14:6397. doi: 10.1038/s41467-023-42211-8 (PMC10618251; doi:10.1038/s41467-023-42211-8)
Supplement: Supplementary file 1 — Supplementary information [file 41467_2023_42211_MOESM1_ESM.pdf]

# Inferring bacterial transmission dynamics using deep sequencing genomic surveillance data

Madikay Senghore [1*], Hannah Read [2], Priyali Oza [2], Sarah Johnson [2], Hemanoel Passarelli-Araujo [1,3], Bradford P Taylor [1], Stephen Ashley [2], Alex Grey [2], Alanna Callendrello [1], Robyn Lee [1,4], Matthew R Goddard [5,6], Thomas Lumley [7], William P Hanage[1], Siouxsie Wiles [2,8*]

**Author affiliations:**
[1]Center for Communicable Disease Dynamics, Department of Epidemiology, Harvard TH Chan School of Public Health, Boston, Massachusetts, USA
[2]Bioluminescent Superbugs Lab, Department of Molecular Medicine and Pathology, University of Auckland, Auckland, New Zealand
[3]Department of Biochemistry and Immunology, Federal University of Minas Gerais, Minas Gerais, Brazil
[4]University of Toronto Dalla Lana School of Public Health: Toronto, Ontario, Canada
[5]School of Biological Sciences, University of Auckland, Auckland, New Zealand
[6]School of Life and Environmental Sciences, University of Lincoln, UK
[7]Department of Statistics, University of Auckland, Auckland, New Zealand
[8]Te Pūnaha Matatini, Centre of Research Excellence in Complex Systems, New Zealand

These authors contributed equally: Madikay Senghore, Hannah Read.
These authors jointly supervised this work: William P Hanage, Siouxsie Wiles.

*Corresponding authors:
Madikay Senghore: msenghore@hsph.harvard.edu
Siouxsie Wiles: s.wiles@auckland.ac.nz

## Tracking the emergence of iSNVs and time to fixation

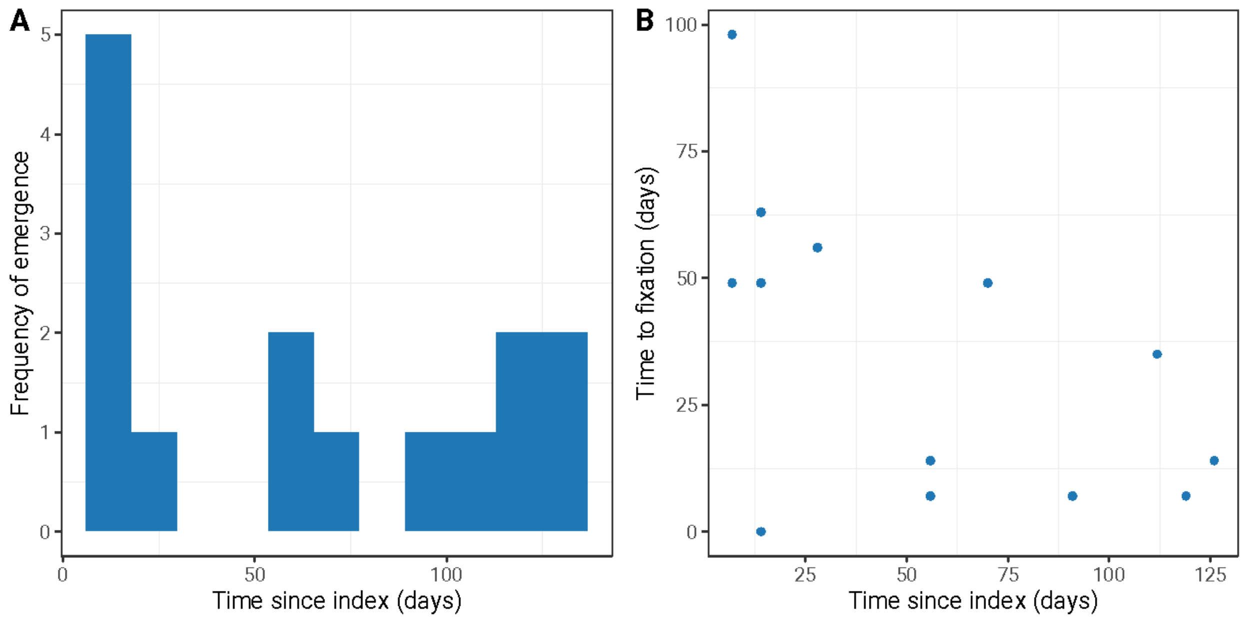


**Supplementary Figure 1. Emergence of iSNVs and the time it takes to become fixed SNVs.**

A) Histogram illustrating the frequency with which iSNVs were emerging at a time t (days) since the index. B) Scatter plot of when iSNVs emerge in the transmission chain and how long it takes them to reach fixation.

## Sharing within host variants is less precise at predicting transmission pairs

We sought to measure the utility of individual loci for inferring transmission pairs based on a variant (allelic frequency > 0.025) being present in tow strains, in other words a shared variant. The Bayes theorem was also used to infer P(T|V), where V represented the number of shared sites that were either an iSNV or a SNV in both isolates and T denotes a transmission event based on the following equaition:

$$P(T|V) = \frac{P(V|T) \times P(T)}{P(V)}$$

On average, strains that were further apart in terms of transmission steps exhibited a lower number of shared variants, although there was some overlap in the distributions (Supplementary Fig 2A). While the mean number of shared iSNVs/SNVs consistently decreased with an increase in transmission steps on average (Supplementary Figure 2B), this alone was insufficient to differentiate transmission pairs from other closely related pairs. Only rarely observed variants (present in less than 5% of the study population) could accurately predict

transmission if they were shared by two isolates (Supplementary Fig 2C). These rare variants often persisted across multiple samples, making them more reliable indicators of whether a secondary isolate belonged to a transmission chain within 5 or fewer transmission steps (Supplementary Figure 2D) (Supplementary Fig 2E).

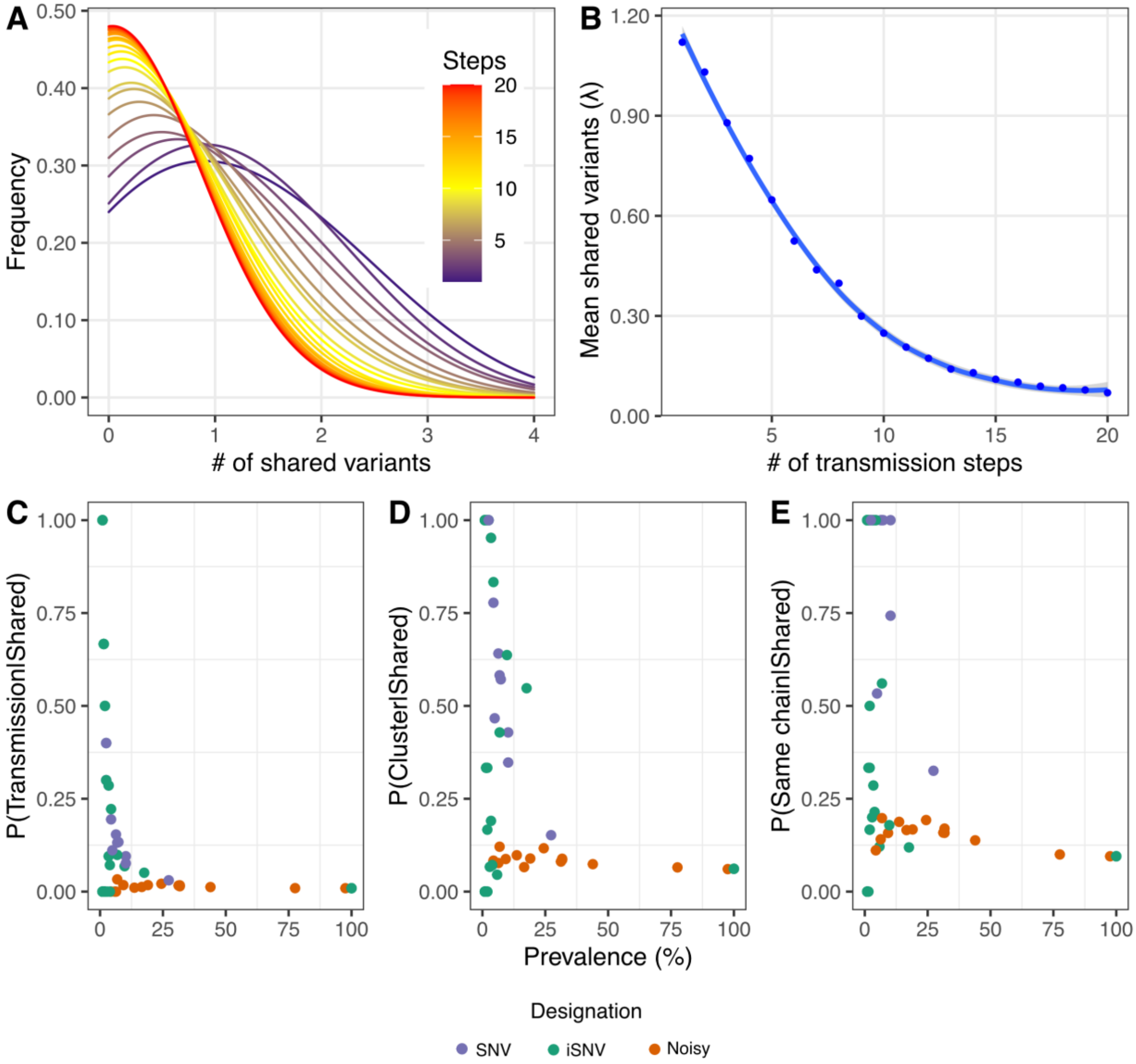


**Supplementary Figure 2. Counting shared genomic variants to infer transmission and the change in the number of shared variants over multiple transmission steps.**

A) Density plot showing the distribution of shared genomic variants, grouped by the number of transmission steps linking pairs. B) The Poisson fitted the mean number of shared variants for pairs linked by the number of transmission steps. C-E) The probability that two isolates are of a transmission pair, from the same transmission cluster or the same transmission chain, given the presence of a variant in both isolates, at the given loci. The x-axis shows the prevalence of variants at the *loci* across the entire dataset.

## Simulating transmission chains with varying bottleneck sizes

This study presents a simulation model that investigates the changes in relative frequencies of within-host variants (iSNVs) during successive transmission steps. The model captures the emergence of iSNVs within a host, their selection dynamics, and the resulting changes in allele frequency (Af) caused by transmission bottlenecks. Additionally, the model considers the possibility of fixed mutations reverting back to iSNVs, subject to selection and changes in Af due to transmission bottlenecks. The simulation framework is based on a previously published model of within-host diversification followed by bottlenecked transmission (Ghafari et al., 2020). To achieve this, we designed a model that simulated transmission chains while allowing for variations in bottleneck sizes. Our model relied on two main assumptions. Firstly, we assumed that prior to transmission, individual single nucleotide variants (iSNVs) experience selection pressure towards fixation, and the resulting change in allelic frequency depends on the initial allele frequency and a constant coefficient, regardless of bottleneck size. Secondly, we postulated that at the point of transmission, iSNVs undergo a secondary change in allelic frequency due to the bottleneck, which can either drive them towards fixation or towards their elimination. Additionally, we considered stochastic emergence of new iSNVs at a constant rate, as well as the possibility of fixed SNVs reverting to iSNVs. Initially, we parameterized our model using 50 variable genomic loci, a *de novo* emergence rate of iSNVs of 0.002 per site per transmission cycle (p), and a selection constant of 3 (S). To further investigate, we explored different combinations of p (0.002, 0.005, 0.01 - representing slow, medium, and fast emergence rates, respectively) and S (1, 3, 5, 10 - representing strong, mild, weak, and very weak selection forces, respectively).

The model incorporates several variables to capture key aspects of the simulation:

- Af: Starting allelic frequency.
- Af': Allelic frequency following selection within the host.
- Af'': New allelic frequency following transmission to a new host via a bottleneck.
- Nb: Size of the bottleneck.
- S: Fixed constant used to calculate changes due to selection.
- N: Total number of variable sites in the genome, representing the finite number of sites where an iSNV can emerge.
- E: Probability of an iSNV emerging at a given site during a transmission.
- R: Probability of a fixed mutation reverting to an iSNV.

**Emergence of Novel iSNVs**: The model represents the emergence of new iSNVs as a two-step process. First, a binomial sampling is performed to determine if a new variant emerges at a specific site.

$$\text{binomial}(n=1, \text{size}=1, \text{prob}=E)$$

Next, the new allelic frequency, AF'', is determined by sampling from a normal distribution. The distribution's parameters are based on the observed mean change in allelic frequency from the Citrobacter dataset.

$$AF'' \sim |N(0.13, 0.10)|$$

**Change in Allelic Frequency at Existing iSNVs**: Prior to transmission, the isolate within a host undergoes selection, resulting in an increase in AF proportional to its value and the fixed strength of selection, S.

$$Af' = (AF + AF(1 - Af)) / S$$

During transmission, the new allele frequency, Af', passes through a bottleneck and assumes a new frequency (Af''). Af'' is normally distributed around Af' with variances determined by the smaller bottleneck sizes.

$$AF'' \sim N(Af', (Af'(1 - Af')) / Nb)$$

**Fixed Mutations Reverting to iSNVs**: The model accounts for the possibility of fixed mutations reverting back to iSNVs through a two-step process. First, a binomial sampling is performed to determine if a fixed mutation reverts to an iSNV. binomial(n=1, size=1, prob=R) Subsequently, the new allelic frequency, AF'', is determined by sampling from a normal distribution, based on the observed mean change in allelic frequency from the Citrobacter dataset.

$$AF'' \sim 1 - |N(0.13, 0.10)|$$

Note: The simulations limit the range of AF' from 0 to 1, recording values below 0 as 0 and values above 1 as 1.

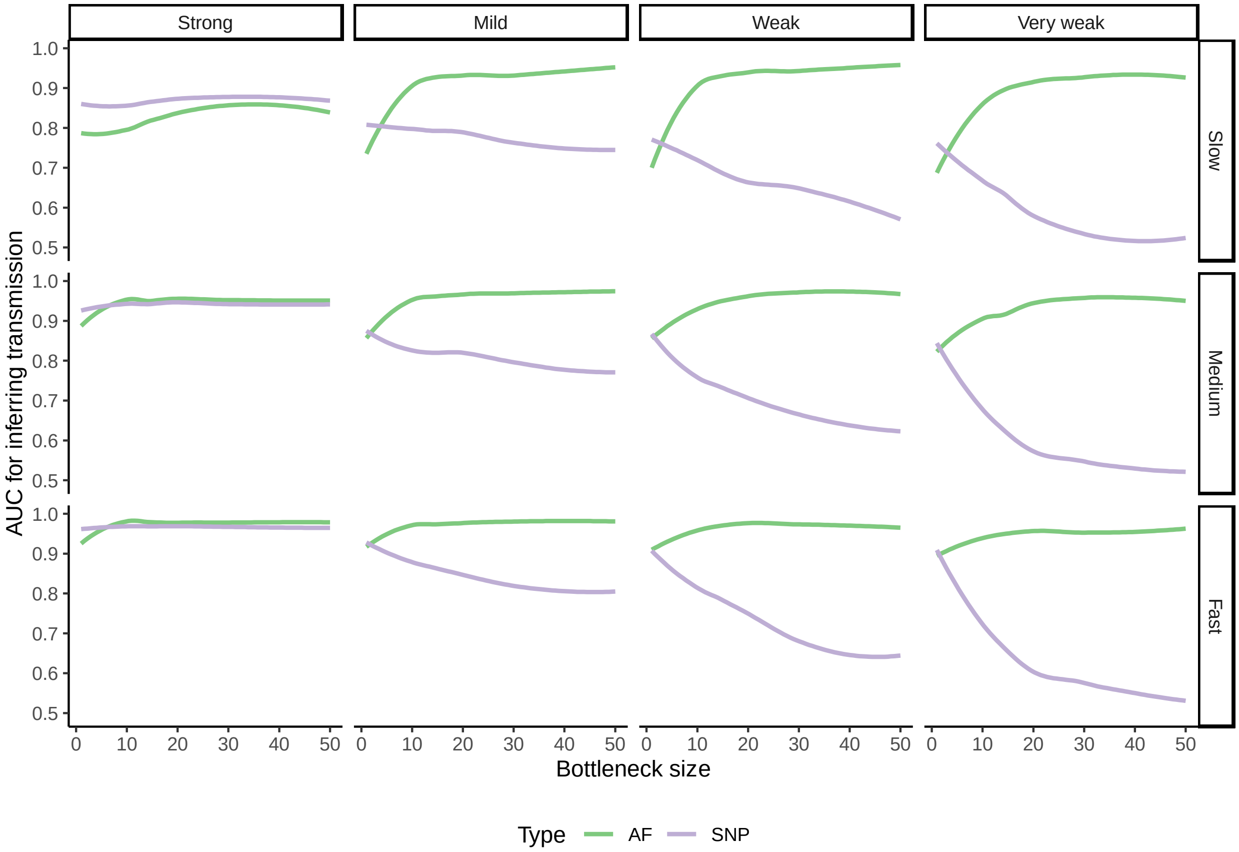


**Supplementary figure 3. Change in allelic frequency infers transmission pairs more effectively at higher bottleneck sizes and is less effective at lower bottleneck sizes when the rate *de novo* emergence of iSNVs is slow.**

The panel figure shows the relationship between bottleneck sizes and the ability of SNP distances and change in allelic frequency to infer transmission pairs, using area under the curve (AUC). Each panel represents a combination of selection parameters and rate of emergence of new iSNVs. Rows correspond to the rate of *de novo* emergence of iSNVs (slow to fast) and the columns correspond to the strength of selection for iSNVs to become fixed (Very weak to strong)
