# Supplementary material for: Inferring bacterial transmission dynamics using deep sequencing genomic surveillance data
Source: Nat Commun. 2023 Oct 31;14:6397. doi: 10.1038/s41467-023-42211-8 (PMC10618251; doi:10.1038/s41467-023-42211-8)
Supplement: Supplementary file 3 — Description of Additional Supplementary Files [file 41467_2023_42211_MOESM3_ESM.pdf]

Inferring bacterial transmission dynamics using deep sequencing genomic surveillance data

Madikay Senghore [1*], Hannah Read [2], Priyali Oza [2], Sarah Johnson [2], Hemanoel Passarelli-Araujo [1,3], Bradford P Taylor [1], Stephen Ashley [2], Alex Grey [2], Alanna Callendrello [1], Robyn Lee [1,4], Matthew R Goddard [5,6], Thomas Lumley [7], William P Hanage[1], Siouxsie Wiles [2,8*]

Author affiliations:
[1]Center for Communicable Disease Dynamics, Department of Epidemiology, Harvard TH Chan School of Public Health, Boston, Massachusetts, USA
[2]Bioluminescent Superbugs Lab, Department of Molecular Medicine and Pathology, University of Auckland, Auckland, New Zealand
[3]Department of Biochemistry and Immunology, Federal University of Minas Gerais, Minas Gerais, Brazil
[4]University of Toronto Dalla Lana School of Public Health: Toronto, Ontario, Canada
[5]School of Biological Sciences, University of Auckland, Auckland, New Zealand
[6]School of Life and Environmental Sciences, University of Lincoln, UK
[7]Department of Statistics, University of Auckland, Auckland, New Zealand
[8]Te Pūnaha Matatini, Centre of Research Excellence in Complex Systems, New Zealand

These authors contributed equally: Madikay Senghore, Hannah Read.
These authors jointly supervised this work: William P Hanage, Siouxsie Wiles.

*Corresponding authors:
Madikay Senghore: msenghore@hsph.harvard.edu
Siouxsie Wiles: s.wiles@auckland.ac.nz

File name: Supplementary Data 1.
Description: This file is comprised of data for each individual animal. Data provided for the route of transmission (oral-gavage or natural transmission); median weight (g); range in weight over the 14-day infection (g); weight change between the start and end of the infection (%); weight change between the start and day 7 of infection (%); bacterial counts prior to co-mingling (colony forming units per gram of stool); calculated area under curve values for bacterial shedding (colony forming units per g stool over the course of the 14-day infection).

File name: Supplementary Data 2.
Description: This file is comprised of data for each single nucleotide variant (SNV). Data provided for position in the genome; base in the reference genome; base in the query sequence; SNV category (downstream gene variant, upstream gene variant, missense variant, stop gained, synonymous variant, non-coding transcript exon variant, splice region variant & non-coding transcript exon variant); effect (low, moderate, high, modifier); gene; locus; accession number; gene type (protein coding, sRNA, pseudogene).
