# Supplementary material for: Inferring bacterial transmission dynamics using deep sequencing genomic surveillance data
Source: Nat Commun. 2023 Oct 31;14:6397. doi: 10.1038/s41467-023-42211-8 (PMC10618251; doi:10.1038/s41467-023-42211-8)
Supplement: Supplementary file 5 — Reporting summary [file 41467_2023_42211_MOESM5_ESM.pdf]

nature portfolio

Corresponding author(s): Siouxsie Wiles & Madikay Senghore

Last updated by author(s): Sep 11, 2023

Reporting Summary

Nature Portfolio wishes to improve the reproducibility of the work that we publish. This form provides structure for consistency and transparency in reporting. For further information on Nature Portfolio policies, see our Editorial Policies and the Editorial Policy Checklist.

Statistics

For all statistical analyses, confirm that the following items are present in the figure legend, table legend, main text, or Methods section.

n/a	Confirmed	
☐	☒	The exact sample size (*n*) for each experimental group/condition, given as a discrete number and unit of measurement
☐	☒	A statement on whether measurements were taken from distinct samples or whether the same sample was measured repeatedly
☐	☒	The statistical test(s) used AND whether they are one- or two-sided<br>*Only common tests should be described solely by name; describe more complex techniques in the Methods section.*
☐	☒	A description of all covariates tested
☐	☒	A description of any assumptions or corrections, such as tests of normality and adjustment for multiple comparisons
☐	☒	A full description of the statistical parameters including central tendency (e.g. means) or other basic estimates (e.g. regression coefficient) AND variation (e.g. standard deviation) or associated estimates of uncertainty (e.g. confidence intervals)
☐	☒	For null hypothesis testing, the test statistic (e.g. *F*, *t*, *r*) with confidence intervals, effect sizes, degrees of freedom and *P* value noted<br>*Give P values as exact values whenever suitable.*
☐	☒	For Bayesian analysis, information on the choice of priors and Markov chain Monte Carlo settings
☒	☐	For hierarchical and complex designs, identification of the appropriate level for tests and full reporting of outcomes
☒	☐	Estimates of effect sizes (e.g. Cohen's *d*, Pearson's *r*), indicating how they were calculated

Our web collection on statistics for biologists contains articles on many of the points above.

Software and code

Policy information about availability of computer code

Data collection	Sequence data were collected by whole genome sequencing on the Illumina platform as described in the methods. In vivo bioluminescence data were collected using an IVIS Kinetic Imaging system and the associated Living Image software (Perkin Elmer)
Data analysis	The code for the sequence data analysis are available via this github repository: https://github.com/msenghore/Citrobacter_manuscript.git. In vivo bioluminescence data were analysed using Perkin Elmer's Living Image software version 4.7.3. Statistical analysis of infection and transmission dynamics data was carried out using the open source lme4 package (version 1.1-27.1) in R (version 4.1) (analysis available). The following bioinformatics software were used: . Trimmomatic (Version 0.35), bwa (version 0.7.17), GATK (version 4.0.2.1), bcftools (version 1.9), Bedtools (version 2.29.2), VCFtools (version 0.1.16), MAFFT (version 7.467), RAxML (version 8.2.12), R (version 4.2.1), and Mega7 (version 7.0.267).

For manuscripts utilizing custom algorithms or software that are central to the research but not yet described in published literature, software must be made available to editors and reviewers. We strongly encourage code deposition in a community repository (e.g. GitHub). See the Nature Portfolio guidelines for submitting code & software for further information.

Data

Policy information about availability of data

All manuscripts must include a data availability statement. This statement should provide the following information, where applicable:

- Accession codes, unique identifiers, or web links for publicly available datasets
- A description of any restrictions on data availability
- For clinical datasets or third party data, please ensure that the statement adheres to our policy

The sequence data have been uploaded to the NCBI short read archives and are available under the project accession number PRJNA884719. The raw infection data have been uploaded to Figshare (https://doi.org/10.17608/k6.auckland.21350790.v1).

Human research participants

Policy information about studies involving human research participants and Sex and Gender in Research.

Reporting on sex and gender	N/A
Population characteristics	N/A
Recruitment	N/A
Ethics oversight	N/A

Note that full information on the approval of the study protocol must also be provided in the manuscript.

Field-specific reporting

Please select the one below that is the best fit for your research. If you are not sure, read the appropriate sections before making your selection.

- [x] Life sciences
- [ ] Behavioural & social sciences
- [ ] Ecological, evolutionary & environmental sciences

For a reference copy of the document with all sections, see nature.com/documents/nr-reporting-summary-flat.pdf

Life sciences study design

All studies must disclose on these points even when the disclosure is negative.

Sample size	In this study we are not seeking to identify a statistically significant difference between transmission chains. Rather, we aimed to determine how many mutational changes occurred between consecutive infections in a transmission chain. To do this, we used 10 transmission chains comprising 200 independent transmission events. In a pilot experiment comprising a single transmission chain, genetic changes were detectable within 10 transmission events. To identify phenotypic changes in transmissibility when compared to Citrobacter rodentium ICC180 in competition with the non-luminescent C. rodentium ICC169, a power size calculation concluded that 5 mice per group was the optimum number of animals needed to obtain statistical significance of $p<0.02$ with 90% power and $p<0.05$ at 95% power. However, we are not reporting on phenotypic changes in transmissibility in this manuscript.
Data exclusions	No data was excluded from the study
Replication	The study was designed with replication embedded within it by including 10 independent transmission chains and 200 independent transmission events. We therefore saw no need for further additional replication.
Randomization	Animals were bred within the Vernon Jensen Unit at the University of Auckland and supplied at 6–7-week-olds in groups of 2 x 5 mice each week. As animals were already from mixed litters, we did not use any specific randomization process to allocate animals to a particular transmission chain or any specific strategies to minimize any confounding factors.
Blinding	Investigators were not blinded during data collection and analysis. This is because animals at data collection were in two different treatment groups with one group needing antibiotic added to their drinking water at regular intervals. Investigators were not blinded at data analysis because the study aimed to determine how many mutational changes occurred between consecutive infections in a transmission chain. In order to determine this we needed to link genomic data to the position in the transmission chain from which the sample was collected.

Reporting for specific materials, systems and methods

We require information from authors about some types of materials, experimental systems and methods used in many studies. Here, indicate whether each material, system or method listed is relevant to your study. If you are not sure if a list item applies to your research, read the appropriate section before selecting a response.

Materials & experimental systems

n/a	Involved in the study	
☒	☐	Antibodies
☒	☐	Eukaryotic cell lines
☒	☐	Palaeontology and archaeology
☐	☒	Animals and other organisms
☒	☐	Clinical data
☒	☐	Dual use research of concern

Methods

n/a	Involved in the study	
☒	☐	ChIP-seq
☒	☐	Flow cytometry
☒	☐	MRI-based neuroimaging

Animals and other research organisms

Policy information about studies involving animals; ARRIVE guidelines recommended for reporting animal research, and Sex and Gender in Research

Laboratory animals	Mus musculus, C57BL/6Elite, 6–7-weeks old, female.
Wild animals	Study did not involve wild animals.
Reporting on sex	Findings apply to one sex only as the experiments were carried out in female mice only. Male mice are more aggressive than females and if not housed together from a young age will fight, leading to stress and injury. As these experiments involved transmission of a mouse pathogen via co-housing of animals of different ages and from different litters, we used female mice only.
Field-collected samples	Study did not involve samples collected in the field.
Ethics oversight	Experiments were approved by the University of Auckland Animal Ethics Committee - application number R1003.

Note that full information on the approval of the study protocol must also be provided in the manuscript.
